# Deutschlands Beteiligung an Einsätzen des UN-*Peacekeeping*. Der Fall MINUSMA

**DOI:** 10.1007/s12399-022-00900-0

**Published:** 2022-06-27

**Authors:** Markus Kaim

**Affiliations:** Stiftung Wissenschaft und Politik, Berlin, Deutschland

**Keywords:** Vereinte Nationen, Peacekeeping, MINUSMA, Mali, Deutsche, Sicherheitspolitik, United Nations, Peacekeeping, MINUSMA, Mali, German security policy

## Abstract

Die militärische Beteiligung an Friedenseinsätzen der Vereinten Nationen gehört zwar rhetorisch zum Kernbestand deutscher Außen- und Sicherheitspolitik. Seit Mitte der 1990er-Jahre hat sich Deutschland jedoch auf eher symbolische Beiträge der Bundeswehr beschränkt. Obgleich die Stärkung der Vereinten Nationen Ziel deutscher Politik bleibt, sind die Motive dafür, sich an Friedensoperationen zu beteiligen, strategisch unterreflektiert. Sich daraus ergebende Zielkonflikte und Dilemmata bleiben ungelöst.

## Abstract (en)

Military participation in United Nations peacekeeping operations is rhetorically part of Germany’s foreign and security policy agenda. Since the mid-1990s, however, Germany has limited itself to rather symbolic contributions by the Bundeswehr. Although strengthening the United Nations remains a goal of German foreign policy, the motives for concrete participation in peace operations are strategically under-reflected. Resulting conflicts of goals and dilemmas remain unresolved.

## Einleitung

Mit der Beteiligung an der United Nations Multidimensional Integrated Stabilization Mission in Mali (MINUSMA) beteiligt sich Deutschland seit 2013 wieder mit einem substanziellen Kontingent an den friedenserhaltenden Operationen der Vereinten Nationen, dem sogenannten *Peacekeeping*. Zuvor hatte sich Berlin für gut 20 Jahre aus diesem Feld zurückgezogen und sich weitgehend auf symbolische Beiträge beschränkt. Es ist zu früh, um zu beurteilen, ob es sich hier um einen Paradigmenwechsel der deutschen Sicherheitspolitik handelt. Aber bemerkenswert ist zumindest, dass diese Umkehr auch programmatisch unterlegt ist. Denn sie entspricht der Prioritätensetzung im *Weißbuch zur Sicherheitspolitik und zur Zukunft der Bundeswehr* aus dem Jahr 2016. Darin wird die Unterstützung der UN im Bereich der Friedenssicherung als zentrale Aufgabe deutscher Sicherheitspolitik definiert.

Auffällig ist jedoch, dass weder das Weißbuch noch andere sicherheitspolitische Dokumente über vage Bekenntnisse hinaus angeben, welchen außenpolitischen Zielen Deutschlands seine Teilnahme an UN-Friedensoperationen eigentlich dienen soll. Damit tut sich eine bemerkenswerte Strategielücke auf – denn hinsichtlich der deutschen Beteiligung an der Gemeinsamen Sicherheits- und Verteidigungspolitik (GSVP) der Europäischen Union und erst recht der Kooperation im Rahmen der NATO geben zahlreiche Dokumente darüber Auskunft, mit welchen Zielen und in welchem Umfang sich Deutschland militärisch in diesen beiden multilateralen Handlungsformaten engagiert.

Grundsätzlich kommt eine Reihe von Motiven in Frage, die Staaten veranlassen können, ein Kontingent für UN-Missionen zu stellen. Eine solche Beteiligung kann militärischer Ausdruck der politischen Solidarität mit Verbündeten sein; sie kann als Ausweis des Einsatzes für die internationale Ordnung und multilaterales Handeln fungieren; mit ihr lässt sich die eigene Handlungsfähigkeit in der internationalen Politik unter Beweis stellen; sie kann die Sicherheitslage in einer strategisch wichtigen Region verbessern, die Folgen der sicherheitspolitischen Entwicklungen in dieser Region für den Entsendestaat abfedern und anderes mehr (Bellamy und Williams 2013).

Häufig lassen sich diese Ziele jedoch weder gleichrangig noch gleichzeitig verfolgen, oft unterliegen sie Beschränkungen durch existierende politische Vorgaben. Mehr noch: Zum Teil schließen sie sich gegenseitig aus. Ohne Klarheit über die Ziele und ihre Priorisierung tut sich eine Leerstelle auf, so dass die sicherheitspolitische Debatte keine Orientierung bieten kann, anhand welcher Kriterien Friedenseinsätze bewertet werden können. Ob Deutschland mit seinem größeren Engagement im UN-*Peacekeeping* – konkret in Mali – erfolgreich ist, liegt daher vollständig im Auge des Betrachters.

Eine besondere Rahmenbedingung kam für die deutsche Außenpolitik im Rahmen der Bewerbung um die zweijährige Mitgliedschaft der Bundesrepublik im UN-Sicherheitsrat 2019/2020 hinzu. Mit dieser Rolle ging die Erwartung einher, dass militärische Beiträge Deutschlands zum UN-*Peacekeeping* eine Verpflichtung seien, die aus dieser nichtständigen Mitgliedschaft im UN-Gremium erwachsen würde. War die Beteiligung an MINUSMA in den Jahren 2019 und 2020 also weltpolitische Pflichtaufgabe und Teil außenpolitischer Staatsräson, ist sie seitdem wieder sicherheitspolitische Kür, die nunmehr stärker als bislang innenpolitischen Kontroversen unterliegt, unter anderem weil die Sicherheitslage in Mali sich seit Jahren verschlechtert hat, in den Jahren 2020 und 2021 zwei Militärputsche im Land stattgefunden haben und die Regierung in Bamako in jüngster Zeit ihre Herrschaft auf die Präsenz russischer Söldner^1^ zu stützen scheint (Thompson et al. 2022). Bundesregierung und Bundestag stehen also im Frühjahr 2022 vor der Aufgabe, nicht nur über die Verlängerung des MINUSMA-Mandats zu befinden, das am 31. Mai 2022 ausläuft, sondern die deutsche Politik in und gegenüber Friedenseinsätzen der Vereinten Nationen und damit einen wichtigen Teil der deutschen UN-Politik neu zu justieren.

Dieser Essay rekapituliert in einem ersten Schritt die Historie der deutschen Beiträge zu friedenserhaltenden Missionen der Vereinten Nationen und arbeitet ihre Treiber ebenso wie ihre Begrenzungen heraus. In einem zweiten Schritt werden die Motive für die Hinwendung der Bundesrepublik zum *Peacekeeping* seit 2013 und das deutsche MINUSMA-Engagement rekonstruiert. Grundlage waren vor allem die Mandatsdebatten im Bundestag aus den Jahren 2013 bis 2021 sowie öffentlich zugängliche Regierungsdokumente. Drittens evaluiert der Beitrag, welche dieser Ziele mit der deutschen MINUSMA-Beteiligung tatsächlich erreicht werden konnten. Abschließend werden mögliche Entwicklungspfade dieser Dimension der deutschen UN-Politik diskutiert.

## Deutsche Beiträge zum UN-*Peacekeeping*

Wie für viele außenpolitische Felder war das Ende des Ost-West-Konflikts und die deutsche Einheit auch für die Frage einer deutschen Beteiligung am UN-*Peacekeeping* eine Wasserscheide (Koops 2016). Denn mit dem *Vertrag über die abschließende Regelung in Bezug auf Deutschland* von 1990 endete die Viermächte-Verantwortung für Berlin und Deutschland als Ganzes und die volle innere und äußere Souveränität des vereinigten Deutschlands wurde hergestellt. In der Folge konfrontierten die Verbündeten Bonn mit der Erwartung, die Bundesrepublik solle nunmehr größere Verantwortung und Lasten in der internationalen Politik jenseits der Bündnis- und Landesverteidigung übernehmen.

In einem Prozess der vorsichtigen Annäherung stellte die Bundesregierung den Vereinten Nationen seit 1989 für mehrere Missionen kleinere Bundeswehrkontingente zur Verfügung, unter anderem für die internationalen Übergangsverwaltungen in Namibia 1989/1990 (UNTAG) und in Kambodscha 1992/1993 (UNTAC). Dabei waren diese Zusagen mit Blick auf die deutsche Öffentlichkeit in doppelter Hinsicht tastend und inkrementell angelegt. In quantitativer, da es sich in allen Fällen um überschaubare deutsche Kontingente handelte. Auch in qualitativer Hinsicht überwog die Vorsicht: Schwerpunkte der deutschen Tätigkeit waren die medizinische Versorgung und die Wahrnehmung polizeilicher Aufgaben. Damit standen diese frühen UN-Missionen mit ihrer humanitären, risikoaversen Orientierung für die Kontinuität eines engen Verständnisses von Bundeswehreinsätzen.

### Die robuste Phase

Die in doppelter Hinsicht zweite Phase der deutschen „Peacekeeping*-*Politik“ begann mit dem UNOSOM-II-Einsatz in Somalia im Jahr 1993: Zum einen verlegte die Bundeswehr mit gut 1700 Soldaten erstmals ein substantielles Kontingent in eine UN-geführte Operation. Damit verließ sie die Praxis früherer Beteiligungen, die in die Kategorie der sogenannten *Token Contributions* fielen, also symbolischer Truppenstellungen, die das Engagement eines Landes in einem Krisengebiet politisch unterstreichen sollen, aber nicht auf militärische Wirksamkeit abzielen.^2^

Zum anderen veränderte sich der Charakter der Einsätze, denn mit der im März 1993 aufgenommenen UNOSOM-II-Mission beteiligte sich die Bundeswehr erstmals an einem „robusten“ Auslandseinsatz im Rahmen einer UN-geführten Operation. Ziel war es, nach dem Ende des somalischen Bürgerkriegs die zwischen den Konfliktparteien vereinbarte Waffenruhe zu überwachen und humanitäre Hilfe für die somalische Zivilbevölkerung zu koordinieren. Zwar bestand der deutsche Unterstützungsverband „nur“ aus einem verstärkten Nachschub- und Transportbataillon. Doch die Sicherheitslage im Land und das Mandat des Sicherheitsrates, das explizit auf Kapitel VII der UN-Charta mit seinen militärischen Zwangsmaßnahmen verwies, ließen keinen Zweifel aufkommen, dass es sich nicht um eine humanitäre Operation traditioneller Prägung handelte.

Ähnliches gilt für die deutschen Beteiligungen an den UN-Operationen auf dem Balkan ab 1995, vor allem an der UNCRO in Kroatien und der UNPROFOR in Kroatien/Bosnien-Herzegowina. Die Bundeswehr stellte jeweils mit mehreren hundert Soldaten substantielle Kontingente, in beiden Fällen operierte sie in einem nicht vollständig befriedeten Umfeld. Alle diese Kontingente dienten verschiedenen Zielen: Sie stärkten das multilaterale Krisenmanagement der Vereinten Nationen und kamen gleichzeitig den gestiegenen Erwartungen westlicher Verbündeter nach, was eine größere sicherheitspolitische Lastenteilung anging; schließlich schufen sie den Rahmen für die sicherheitspolitische „Normalisierung“ Deutschlands.

### Die Abkehr von UN-Einsätzen

Zwei Gründe waren dann dafür verantwortlich, dass die Bundesrepublik sich vom aktiven Engagement im UN-*Peacekeeping* abgewendet hat: Erstens war sie, wie andere westliche Verbündete ebenfalls, enttäuscht über die begrenzte militärische und politische Wirkung von UN-Operationen. Zweitens präferierte sie die seit den 1990er-Jahren entstandenen alternativen Foren für eine deutsche Beteiligung am internationalen Krisenmanagement, nämlich die NATO (u. a. mit KFOR und ISAF) und die EU (u. a. mit EUFOR Althea und EUFOR RD Congo). So blieb der maritime UNIFIL-II-Einsatz (seit 2006) über Jahre der einzige substantielle Ausweis deutschen Engagements im UN-*Peacekeeping*.

Mit der deutschen Beteiligung an der MINUSMA-Operation in Mali scheint vordergründig ein erneuter Paradigmenwechsel erkennbar. Seit Juli 2013 beteiligt sich die Bundeswehr an dieser multidimensionalen Mission, die zwei Hauptaufgaben verfolgt, nämlich, erstens, die Unterstützung der malischen Regierung bei der Umsetzung des Friedensabkommens mit den separatistischen Tuareg-Rebellen im Norden von Mali sowie, zweitens, den Schutz der Zivilbevölkerung und die Wiederherstellung der staatlichen Autorität im Zentrum des Landes. Daneben soll MINUSMA vertrauensbildende Maßnahmen unterstützen, die Einhaltung der Menschenrechte überwachen und humanitäre Hilfsleistungen absichern. Gemäß dem Mandat des Bundestages können bis zu 1100 Bundeswehrsoldaten in Mali bzw. der Region als Teil des mehr als 12.000 Soldaten umfassenden UN-Kontingents stationiert werden. Mit 970 Soldaten (Stand: 17. Januar 2022) ist diese Option zurzeit jedoch bei Weitem nicht ausgereizt.

## Die Motive für das militärische Engagement Deutschlands in Mali

Geht man der Frage nach, wie die Entscheidung zur MINUSMA-Beteiligung getroffen und begründet wurde, drängt sich der Eindruck auf, dass sich an dem tradierten außen- und sicherheitspolitischen Muster der deutschen Zurückhaltung nichts Wesentliches geändert hat. Und auch die dürftige Reflexion über das mit MINUSMA angestrebte politische Ziel steht in einer langen Tradition der Befassung des Bundestages mit Auslandseinsätzen der Bundeswehr. Dominierten zu Beginn des Einsatzes vor allem „krisenferne“ Motive, die mit der Situation in Mali und der Sahel-Region nichts zu tun hatten, traten mit zunehmender Dauer des Einsatzes landes- und regionalspezifische Motive in den Vordergrund.

### Bündnissolidarität

Deutschland hat das militärische Engagement in Mali nicht aktiv gesucht, sondern hat dem Wunsch eines wichtigen Verbündeten entsprochen – wie eigentlich alle größeren Auslandseinsätze der Bundeswehr nach diesem Muster zustande gekommen sind. Waren im Fall des Afghanistan-Engagements die USA der Auslöser, genauer gesagt deutsche Überlegungen zur transatlantischen Solidarität nach den Terroranschlägen vom 11. September 2001, so drängte im Jahr 2013 Paris die Bundesrepublik zu einem Einsatz in Mali an der Seite Frankreichs. Wie im Fall Afghanistan waren Überlegungen bezüglich des landes- oder regionalspezifischen Krisenmanagements zu Beginn der Operation von untergeordneter Bedeutung.

Schnell machte die französische Regierung unter Präsident François Hollande ihre Position klar: Zwar habe Frankreich aus der Not heraus die Führung dieses Einsatzes übernommen, erwarte aber, dass sich andere europäische Länder anschlössen. Einerseits sollten sie Paris militärisch unterstützen und der französischen Vorgehensweise politische Legitimität verschaffen, andererseits mit ihrer Beteiligung die gewachsene sicherheitspolitische Rolle Europas illustrieren.^3^ Für diesen europäischen Weg (unter Pariser Führung) benötigte Frankreich Deutschland als entscheidenden Partner. Die Unterstützung vonseiten kleinerer EU-Mitgliedstaaten war zwar ebenfalls willkommen, aber nur die Beteiligung Berlins versprach, die angestrebte doppelte politische Signalwirkung zu entfalten.

Dieses zweifache Motiv der Bündnissolidarität und der Manifestation eines europäischen Fußabdrucks wurde zum eigentlichen Movens der deutschen Politik hinsichtlich MINUSMA.^4^ Landes- oder regionalspezifische Ordnungsvorstellungen traten dahinter zurück: Deutschland hat Paris in diesen Fragen die Federführung überlassen und sich aus diesbezüglichen Planungen weitgehend herausgehalten.

Erkennbar ist das angesprochene Doppelmotiv weniger an den jeweiligen Mandatierungen durch den Deutschen Bundestag seit 2013, da diese vor allem rechtlichen und militärischen Erwägungen Rechnung tragen müssen. Doch die begleitenden Reden, insbesondere diejenigen der Vertreter der Regierungsfraktionen, illustrieren den Punkt sehr deutlich, indem sie in der einen oder anderen Form auf das französische Eingreifen und die Notwendigkeit deutscher Solidarität mit Paris verweisen. In den folgenden Jahren trat das Motiv der Bündnissolidarität in den politischen Begründungen für die deutsche MINUSMA-Beteiligung etwas in den Hintergrund, ohne jedoch vollständig an Bedeutung zu verlieren. Erst seit dem Amtsantritt von Frankreichs Präsident Emmanuel Macron im Mai 2017 zieht es sich wieder stärker durch die Argumentation der Bundesregierung, da die französische Regierung ihre Erwartungen an Berlin offensiver vorträgt, nicht zuletzt, weil die französische Malipolitik kaum Erfolge zeitigt.

Die Erkenntnis, dass sich das Engagement der Bundesrepublik in Westafrika politisch vor allem aus den Erwartungen Frankreichs speist, hat zwei Dimensionen: So lautet die Frage nicht nur, ob Deutschland überhaupt an MINUSMA teilnimmt, sondern die zweite Frage des „Wie“ ist damit eng verbunden. Das heißt, die jeweiligen Ordnungsvorstellungen Frankreichs für Mali bzw. den Sahel definieren den Rahmen der deutschen Politik in dieser Region.

Dabei können die prinzipiellen Bekundungen zur deutsch-französischen Zusammenarbeit im Sahel nur schwer verdecken, wie weit Paris und Berlin bei der konkreten Ausgestaltung dieser Kooperation auseinanderliegen. In Paris wird das deutsche Engagement in MINUSMA zwar grundsätzlich geschätzt, aber wie ein roter Faden ziehen sich französische Vorbehalte durch die bilaterale Sahel-Politik. Sie betreffen einerseits den Umfang des Engagements, andererseits die Einsatzregeln der jeweiligen Kontingente. Zum Beispiel hat sich der Eindruck verfestigt, das Sicherheitsrisiko für Bundeswehrsoldaten solle auf ein absolutes Minimum reduziert werden. Denn Terrorismusbekämpfung betrieb die Bundeswehr allenfalls mittelbar. Oberste Priorität genoss die Kontingente sicher durchzuschleusen, weswegen ihre Raumpräsenz und ihre Interaktionen mit der Bevölkerung äußerst begrenzt blieben.

Diesen Eindruck hat die Bundesregierung ungewollt dadurch verstärkt, dass Berlin in der Legislaturperiode 2017–2021 lange gezögert hat, sich intensiver als bislang zu engagieren. Ende 2019 hat die Bundesregierung lediglich politische Unterstützung für die Taskforce Takuba zugesagt, mit der eine Gruppe von elf europäischen Staaten – Deutschland, Belgien, Dänemark, Estland, Frankreich, Norwegen, die Niederlande, Portugal, Tschechien, Großbritannien und Schweden – in einer informellen Koalition der Willigen den Kampf gegen Dschihadisten in Mali mit Spezialkräften unterstützen will.

Dieser immer wiederkehrende Dissens geht indes tiefer und reflektiert unterschiedliche politische und militärische Prämissen, etwa bezüglich der Frage, in welchem Umfang militärische Gewalt angewendet werden solle und welchen Nutzen sie habe:

Wenn Deutschland und andere sich nicht an robusten Mentoring-Einsätzen der von Paris initiierten Task-Force *Takuba* im Sahel beteiligen, dann liegt das nicht ausschließlich an Risikofeindlichkeit, wie viele in Paris vermuten, sondern auch an der Vorstellung in Berlin, dass der Einsatz von mehr Militär kein aussichtsreicher Weg ist. […] Hier treten Unterschiede der strategischen Kultur zutage, die auf beiden Seiten Unverständnis und Frustrationen fördern (Tull 2020, S. 2).

Das wirft für Deutschland die Frage auf, wie effektiv die deutschen Bemühungen zur Unterstützung Frankreichs tatsächlich sind und wie kraftvoll das damit verbundene Signal der Bündnissolidarität sein kann. Vor diesem Hintergrund ist die Beobachtung wichtig, dass in Frankreich die Zustimmung für den Militäreinsatz mittlerweile bröckelt: Trotz des seit 2013 verfolgten Ziels, einerseits den islamistischen Terrorismus einzudämmen und andererseits das Land bzw. die Region zu stabilisieren, hat die politische Instabilität und Unsicherheit angehalten und sogar noch zugenommen. Angesichts dieser Entwicklung hat Präsident Macron im Juni 2021 das Ende der Operation Barkhane in Mali angekündigt. Von den 5100 französischen Soldaten solle bis 2023 ein Großteil abgezogen werden. Frankreich werde stattdessen nach einem „anderen Format“ für seine militärische Präsenz im Land suchen. Im Januar 2022 hat Frankreichs Außenminister Jean-Yves Le Drian der malischen Militärjunta einen doppelten Vertrauensbruch vorgeworfen. Sie halte sich nicht mehr an das Versprechen eines demokratischen Übergangs, da sie die angestrebten Wahlen auf das Jahr 2025 verschoben hat. Zudem stelle sie mit Flugverboten und einer Fülle weiterer operativer Einschränkungen sowohl die französische als auch die europäische militärische Unterstützung in Frage. Die Bedingungen für eine Fortführung der internationalen Militärpräsenz seien damit nicht länger gegeben (Wiegel 2022).

Lediglich mutmaßen lässt sich über die Folgen dieser Entscheidung für die deutsche Malipolitik: Nimmt man das Solidaritätsargument ernst, wäre es wahrscheinlich, dass mit einem französischen Rückzug aus Mali ebenso das deutsche MINUSMA-Engagement sehr schnell an ein Ende käme. Diese Situation findet ihren Ausdruck in dem auch aus anderen Auslandseinsätzen bekannten Diktum „Gemeinsam rein, gemeinsam raus“. Jüngst haben bereits Außenministerin Annalena Baerbock und die Vorsitzende des Verteidigungsausschusses, Marie-Agnes Strack-Zimmermann, ihrer Skepsis Ausdruck verliehen, ob der Mali-Einsatz noch die angestrebten politischen Ziele erreichen könne (Hemicker 2022).

### Münchner Konsens

Ein zweiter Treiber für das deutsche Engagement im Rahmen von MINUSMA ist die sogenannte Verantwortungsdebatte bzw. ihr vorläufiges Ergebnis, der „Münchner Konsens“. Auf der Münchner Sicherheitskonferenz von 2014 hatten Bundespräsident Joachim Gauck, Außenminister Frank-Walter Steinmeier und Verteidigungsministerin Ursula von der Leyen in gleichgerichteten Reden gefordert, Deutschland solle angesichts des veränderten sicherheitspolitischen Umfelds mehr Verantwortung in der internationalen Politik übernehmen. Häufig als Aufruf ausschließlich zu einem größeren militärischen Engagement missinterpretiert, zielten diese Reden vor allem darauf, die europäische Nachbarschaft aktiver politisch zu gestalten und diejenigen Institutionen zu stärken, die den multilateralen Handlungsrahmen der deutschen Außenpolitik bilden.^5^ Eine Dimension dieses Postulats bestand darin, sich intensiver als bisher bei den UN zu engagieren. Niederschlag fand das beispielsweise für den Bereich der UN-Friedensmissionen beim Leaders’ Summit on Peacekeeping 2015 in New York und auf dem UN Peacekeeping Defence Ministerial 2016 in London. Bei beiden Gelegenheiten bekannte sich die Bundesregierung zu einem aktiven Engagement (Kaim und Strauß 2017).

Auch das sicherheitspolitische Weißbuch der Bundesregierung von 2016 griff Friedensmissionen als Handlungsfeld auf und platzierte die UN zum ersten Mal an prominenter Stelle unter den multilateralen Organisationen, in denen Deutschland sicherheitspolitisch tätig ist:

Es ist deshalb unser vorrangiges Ziel, das System der VN durchsetzungsfähiger zu machen, die VN weiter zu stärken und zur effizienteren Wahrnehmung ihrer Aufgaben zu befähigen. Deutschland wird weiterhin dazu beitragen, die VN für die zunehmend komplexer und vielschichtiger werdenden Aufgaben aufzustellen, indem insbesondere […] das deutsche Engagement, unter anderem durch Stärkung materieller und personeller Beiträge zu und Übernahme von Führungsverantwortung in VN-Missionen (zivil, polizeilich, militärisch) und im VN-Sekretariat, ausgebaut wird; Missionen der VN mit zivilen und militärischen Hochwertfähigkeiten unterstützt werden (Bundesministerium der Verteidigung 2016, S. 63).

Ausdrücklicher als in früheren Dokumenten und Äußerungen definiert die deutsche Außenpolitik seitdem neben den beiden traditionellen außenpolitischen Handlungsformaten NATO und EU nunmehr auch ihr Engagement in den Vereinten Nationen, mit dem sie hervorhebt, dass sie regional wie global mehr Verantwortung zu übernehmen bereit ist.

Im sogenannten Münchner Konsens gipfelt eine Entwicklung, die im Kern früher begonnen hat. Denn bereits seit 2012 wuchs in der Bundesregierung die Einsicht, dass Deutschland aktiver und engagierter die internationalen Beziehungen gestalten müsse. So bezeichnete sie Deutschland in dieser Zeit als eine von zahlreichen „Gestaltungsmächten“ und bekundete nach außen einen stärkeren Willen, sich global einzubringen. Ein Beispiel für die Umsetzung desselben war von Beginn an die Afrikapolitik der Bundesregierung. Die Beteiligung an der UN-Mission in Mali ist in diesem Kontext immer wieder als Zeichen für den neuen Willen, Verantwortung zu tragen, genannt worden (Junk 2014).

Als Präsident Gauck 2014 seine Rede hielt, hatte Russland die Krim noch nicht annektiert, die Flüchtlingskrise 2015 hatte die EU noch nicht gespalten, Donald Trump war noch nicht Präsident der USA und der Austritt Großbritanniens aus der EU noch nicht beschlossen. Auch die Konkurrenz der drei Großmächte USA, China und Russland hatte noch nicht jene konfrontativen Konturen gewonnen, die heute die Weltpolitik bestimmen. Kurz: Große Teile der internationalen Ordnung im machtpolitischen, institutionellen und normativen Sinne waren noch nicht diesem starken Wandel unterworfen, der seitdem erkennbar ist. Diese Umwälzungen in der internationalen Politik haben zu einer radikal veränderten Ausgangslage für die deutsche Außenpolitik geführt, die das Argument der größeren internationalen Verantwortung noch schwerwiegender macht. 2014 ging es für die deutsche Außenpolitik vor allem darum, den Erwartungen der Partner und Verbündeten gerecht zu werden. 2021 gilt es vorwiegend, sich in einer Welt zu behaupten, in der nicht länger eindeutig zuzuordnen ist, wer noch Partner und Verbündeter ist und wer nicht mehr, und in der die Institutionen und Handlungsmaximen multilateralen Handelns von verschiedenen Seiten zunehmend in Frage gestellt werden. Ein funktionierendes multilaterales System ist unabdingbare Voraussetzung dafür, dass Deutschland und Europa international Einfluss nehmen können. Angesichts dessen speist sich das Engagement Berlins für den Erhalt und die Weiterentwicklung multilateraler Prinzipien einerseits aus den skizzierten Überlegungen des Münchner Konsenses, andererseits ist es Ausdruck eines aufgeklärten Eigeninteresses daran, dass die internationale Ordnung funktioniert.^6^ In diesem Sinne ist also auch Deutschlands Engagement für die internationale Zusammenarbeit in multilateralen Institutionen unter dem Titel „Allianz für den Multilateralismus“ im Kern eine Fortführung des Münchner Konsenses bzw. eine Ausformung desselben (Speck 2018).

### Der Sitz im Sicherheitsrat

Zeitgleich zu ihrer Beteiligung an MINUSMA bewarb sich die Bundesrepublik um einen nichtständigen Sitz im UN-Sicherheitsrat für 2019/2020. Um eine ernsthafte Chance auf den Sitz zu haben, war es notwendig, dass Deutschland versprach, seine Beiträge zu den Friedensoperationen der UN substantiell und auf mehreren Ebenen – politisch, strategisch, operativ – auszubauen und neu zu strukturieren. Von dieser Einsicht ließ sich das Auswärtige Amt bereits leiten, als es die Friedenssicherung als einen von vier Schwerpunkten der deutschen Bewerbung hervorhob.

Für die Bewerbung um den nichtständigen Sitz, die der damalige Außenminister Frank-Walter Steinmeier im Juni 2016 offiziell bekanntgab, machte sich die Bundesregierung zudem die angesprochene Debatte um Verantwortung gezielt nutzbar. Sie kontrastierte dabei die eigene Bereitschaft zu größerem Engagement mit der Krise, in der das multilaterale System sich befände. Bereits während der Kampagne für einen Sitz im Sicherheitsrat 2011–2012 hatte Deutschland zudem versprochen, sich in Fragen der afrikanischen Sicherheit zu engagieren. Vor diesem Hintergrund stand nun die Erwartung im Raum, dass Deutschland auch 2019–2020 einen konkreten politischen und/oder militärischen Beitrag zu den afrikanischen Dossiers im Sicherheitsrat leisten müsse. Einige Beobachter leiteten aus diesen Äußerungen sogar eine mögliche Prioritätensetzung für die deutsche Ratsagenda ab: Berlin solle versuchen, eine größere Rolle in den Ratsdebatten über die UN-Friedensoperationen in Afrika zu spielen, denn diese nähmen mehr als die Hälfte der Zeit des Sicherheitsrates in Anspruch und forderten den weit überwiegenden Teil der eingesetzten Soldaten und Polizisten (Gowan 2018).

Deutschland wurde am 8. Juni 2018 mit 184 von 190 möglichen Stimmen in den UN-Sicherheitsrat 2019–2020 gewählt. In dem sehr guten Abstimmungsergebnis manifestierten sich die hohen Erwartungen an Deutschlands Engagement im wichtigsten politischen Gremium der Vereinten Nationen im Allgemeinen ebenso wie in Form seiner konkreten Aktivitäten im Speziellen. Sofern über eine größere Rolle Europas im UN-*Peacekeeping* nachgedacht wird, steht Deutschland im Mittelpunkt auch dieser Überlegungen.

### Europäische Handlungsfähigkeit

Die deutsche Beteiligung an MINUSMA ist als Teil einer breiteren Bewegung zu betrachten, nämlich eines „European return to UN Peacekeeping“ (Koops 2016, S. 652). Einige europäische Staaten, die in früheren Dekaden substantielle Beiträge zu den UN-Truppen geleistet, dann aber aus einer Reihe von Gründen ihr Engagement erheblich reduziert haben, kehren vorsichtig in UN-Friedenseinsätze zurück – Dänemark, Frankreich, Irland, Italien, Norwegen, Schweden, die Niederlande und Großbritannien. Wie bereits angedeutet, ist dies unter anderem dem Streben nach einer größeren außen- und sicherheitspolitischen Handlungsfähigkeit der EU geschuldet. So weist die European Union Global Strategy (EUGS) – wie auch das deutsche sicherheitspolitische Weißbuch von 2016 – der europäischen Unterstützung für UN-Friedensoperationen eine zentrale Bedeutung zu.

Dabei fügt es sich, dass die EU in den vergangenen fünfzehn Jahren bereits diverse friedenserhaltende Operationen nach Afrika entsandt hat, mehr als jede andere internationale Organisation, einschließlich der UN und afrikanischer Regionalorganisationen. Damit hat sie im doppelten Sinne wichtige Erfahrungen gewonnen, nämlich einerseits als eigenständiger Akteur im Feld des *Peacekeeping* mit seinen komplexen politischen und militärischen Herausforderungen, andererseits gerade in demjenigen geografischen Umfeld, in dem die meisten Friedensoperationen der Vereinten Nationen stattfinden (Unser 2014, S. 572–574).

Darüber hinaus stellen die EU-Mitgliedstaaten bis zu 40 % des UN-Friedenssicherungsbudgets und die Union unterstützt afrikanische Regionalorganisationen finanziell durch die African Peace Facility (APF). Seit der Entsendung ihrer ersten Friedensmission hat sich die EU zu einem maßgeblichen Akteur für die Friedenssicherung auf dem afrikanischen Kontinent entwickelt, ohne dessen Unterstützung die meisten Operationen afrikanischer Organisationen nicht durchgeführt worden wären (Scheuermann 2012, S. 99–101). Ob sie damit jedoch das UN-*Peacekeeping* auf Dauer gestärkt hat, ist strittig: Denn mit der Entwicklung der GSVP verfügen die EU-Mitglieder zugleich über ein ausschließlich von ihnen kontrolliertes multilaterales Format – eine vordergründig attraktivere Alternative zu einer direkten Beteiligung an friedenserhaltenden Missionen der UN.

Auffällig ist, dass es sich bei den EU-Missionen überwiegend um sehr speziell zugeschnittene Operationen handelt, die sich von den groß angelegten, umfassenden Missionen der UN unterscheiden. In vielen Fällen besetzt die EU funktionale Nischen, die die Vereinten Nationen nicht anderweitig füllen können, und spielt somit eine unterstützende Rolle innerhalb klar definierter Tätigkeitsfelder. Dieser von den Europäern gewählte Nischenansatz basiert weitgehend auf Präferenzen der EU bzw. ihrer Mitgliedstaaten (Brosig 2014, S. 75). Die Motivation für die Entwicklung dieses funktionalen Nischenansatzes bei der Friedenssicherung ist leicht zu identifizieren. Er zielt darauf ab, die bekannten Schwächen von UN-Einsätzen soweit möglich zu vermeiden, etwa die schleichende Veränderung des Mandats, die Übernahme von Hochrisikoeinsätzen in scheinbar aussichtslosen Konflikten, die Entsendung von Missionen ohne klare zeitliche Begrenzung oder das Problem von zu ehrgeizigen Mandaten (Tardy 2019).

Damit prägt die Art der Truppensteller die Art der Fähigkeiten, die den UN bzw. der EU zur Verfügung gestellt werden. Theoretisch könnten UN-Einsätze von den militärischen Fähigkeiten europäischer Staaten profitieren, wenn Letztere substantieller und dauerhaft zu UN-Einsätzen beitrügen. Die trotz der skizzierten „Rückkehr“ nach wie vor dünne Präsenz Europas – und der meisten Länder mit hochtechnologischer militärischer Ausrüstung – von UN-Einsätzen entzieht diesen jedoch die Ausrüstung, auf die EU- oder NATO-Einsätze derweil zurückgreifen können.

Um die spezialisierten Fähigkeiten der EU effektiver zu nutzen, ist eine systematischere Form der Zusammenarbeit zwischen ihr und der UN notwendig. Parallel zur Kooperation vor Ort haben beide Organisationen daher ihre Beziehungen zunehmend institutionalisiert, beginnend mit zwei gemeinsamen Erklärungen 2003 und 2007, die gemeinsame Kommunikationskanäle und Koordinationsmechanismen festlegen. So sind zum Beispiel zwei Treffen pro Jahr des UN-EU Steering Committee on Crisis Management vereinbart worden und die beiden Abteilungen der UN für Friedensoperationen und politische Angelegenheiten haben ein Verbindungsbüro in Brüssel eröffnet. Der Plan of Action to Enhance EU CSDP Support to UN Peacekeeping von 2012 sowie die UN-EU Strategic Partnership on Peacekeeping and Crisis Management von 2015 haben schließlich eine Reihe von Kooperationsfeldern definiert, die für friedenserhaltende Maßnahmen von zentraler Bedeutung sind: schnelle Eingreiffähigkeiten, die Unterstützung der Afrikanischen Union (AU) in diesem Politikfeld, größere Beiträge von EU-Mitgliedern zu UN-Missionen, Kooperation im Bereich der Rechtsstaatsentwicklung und der Reform des Sicherheitssektors, Zusammenarbeit in logistischen Fragen und nicht zuletzt einen besseren Austausch von Informationen und Analysen (Pietz 2013).

Den grundlegenden politischen Rahmen für das Engagement der EU zugunsten von *Peacekeeping* und *Peacebuilding* bildet die EUGS. So will Brüssel nicht nur EU-Missionen vor Ort besser mit UN-Missionen koordinieren, sondern auch generell die Kooperation mit den UN intensivieren. Vor diesem Hintergrund haben die Vereinten Nationen und die EU im September 2020 am Rande der UN-Generalversammlung ein Abkommen unterzeichnet, das die Zusammenarbeit verbessern und die gemeinsame Reaktion bei Friedenseinsätzen und im Krisenmanagement stärken soll. Das Rahmenabkommen zwischen der Europäischen Union und den Vereinten Nationen über gegenseitige Unterstützung bei ihren jeweiligen Missionen und Operationen vor Ort soll die operative Abstimmung erleichtern und die Komplementarität zwischen EU- und UN-Feldmissionen in den Bereichen Logistik, medizinische Versorgung und Sicherheitsunterstützung erhöhen.

### Die Sicherheit der „Anderen“ – das Motiv der regionalen Sicherheit

Dominierte bei der Erstmandatierung des Bundeswehreinsatzes in Mali im Jahr 2013 das Argument der Bündnissolidarität, traten erst anschließend Erwägungen zur sicherheitspolitischen Entwicklung in Mali und der Sahel-Region hinzu und zum Teil in den Vordergrund, wie sich an den Mandatsdebatten im Deutschen Bundestag zeigen lässt. Dabei mangelt es in den entsprechenden Beiträgen zuweilen an spezifischen Ausführungen zur (angespannten) Sicherheitslage in der Region. Viele Redner bringen stattdessen die deutsche MINUSMA-Beteiligung allgemein mit deutscher Afrikapolitik in Verbindung und setzen die Entsendung des Bundeswehrkontingents in den Kontext eines eher zivilen, entwicklungspolitisch begründeten Engagements. Die Breite des MINUSMA-Mandats erleichtert diesen Blickwinkel. Diese argumentative Verknüpfung wird noch verstärkt durch einen Verweis, der sich wie ein roter Faden durch die Debatten zieht, dass nämlich MINUSMA Teil des sogenannten vernetzten Ansatzes sei, das heißt des deutschen Grundprinzips im internationalen Krisen- und Konfliktmanagement.

Die Bundestagsdebatten illustrieren zudem, dass denjenigen Abgeordneten, die das Mandat befürworten, ebenso wie den Vertretern der Bundesregierung durchaus klar ist, dass die Bilanz des Erreichten bescheiden ausfällt. Spätestens seit 2018 drücken die MINUSMA-Befürworter in den Mandatsdebatten immer wieder ihre Unzufriedenheit aus – über die Zunahme terroristischer Anschläge, über die mangelhafte Umsetzung des Friedensabkommens, über die allgemeine Verschlechterung der Sicherheitslage, schließlich über die fehlende Bereitschaft der in Mali herrschenden Eliten, ausreichend Verantwortung zu übernehmen, damit sich Staatlichkeit herausbilden kann.

Fraglich erscheint, welchen Beitrag Deutschland sowie die Vereinten Nationen überhaupt leisten können angesichts derjenigen politischen Rahmenbedingungen, unter denen sie in Mali zu operieren haben. Landes- und Regionalexperten sehen in dieser Frage nur geringe Handlungsspielräume und konstatieren, die Aufrechterhaltung des Status quo biete ein Gleichgewicht, mit dem sowohl die internationale Gemeinschaft als auch die malische Regierung leben können (Tull 2019, S. 420–421).

### Die „eigene“ Sicherheit – Migration und Terrorismus

Neben dem Motiv der regionalen Sicherheit (s. Kap. 3.5) wird in der Debatte im Deutschen Bundestag ein weiteres Motivbündel als Begründung für die deutsche MINUSMA-Beteiligung angeführt. Mit dem Einsatz werde nicht nur die Sicherheitslage im Sahel verbessert, sondern er diene insofern auch deutschen Interessen, als dass er zwei sicherheitspolitische Herausforderungen aus der Region abmildere, die Deutschland bzw. Europa direkt beträfen: Zum einen drohten ohne MINUSMA Migrationsströme aus dem und durch den Sahel nach Europa zu erstarken. Zum anderen sei ein Ausgreifen islamistischer Gruppen aus Mali nach Europa zu befürchten. Entsprechende Anschläge innerhalb der EU könnten die Folge sein. In Variation eines bekannten, anders gelagerten Zitats ließe sich dieses Argument unter dem Titel fassen „Deutschlands Sicherheit wird auch im Sahel verteidigt“.

Insbesondere seit der „Migrationskrise“ 2015 und den Terroranschlägen von Paris im November 2015 sind die MINUSMA-Debatten im Deutschen Bundestag um diese beiden Facetten ergänzt worden. Selbst wenn manche Stimmen seit Jahren davor warnen, die multiplen Konfliktlagen in Mali vor allem auf eine terroristische Herausforderung zu reduzieren, so besteht doch wenig Zweifel, dass gerade diese Sicht neben der Migrationsfrage seitdem ein wichtiger Grund für die Bundesrepublik ist, sich in Mali zu engagieren.

## Bewertung der Motive

Dieser Aufsatz hat sechs „Motivbündel“ identifiziert, die dazu geführt haben, dass – nach langer Abstinenz – zum ersten Mal wieder ein größeres deutsches Kontingent als Teil einer UN-geführten Friedensoperation entsandt worden ist bzw. das entsprechende Mandat immer wieder verlängert worden ist. Abschließend ist zu fragen, welche dieser Ziele mit der deutschen MINUSMA-Beteiligung erreicht werden konnten.

1. Bündnissolidarität: Dieses Argument basiert auf der Annahme, die Präsenz der Bundeswehr in Mali (MINUSMA und EUTM) sei Ausweis deutscher Solidarität mit Paris – trotz existierender rechtlicher und operativer Einschränkungen und unabhängig von französischen Forderungen nach einem robusteren Engagement. Sie stärke somit Deutschlands Ansehen als „verlässlicher Partner“ in den Kreisen seiner sicherheitspolitischen Verbündeten und im Rahmen des internationalen Krisenmanagements. Für diese Form der Unterstützung dürfe Deutschland seinerseits ein Entgegenkommen in anderen Politikfeldern erwarten.

Ob die MINUSMA-Beteiligung tatsächlich dem erklärten deutschen Ziel dient, die Solidarität mit Frankreich wirksam unter Beweis zu stellen, muss bezweifelt werden, vor allem je länger der Einsatz dauert. Unstrittig ist, dass dieses Anliegen gerade zu Beginn das deutsche Handeln geleitet hat, und es wäre unfair, den handelnden Akteuren zu unterstellen, sie hätten bei der Erstmandatierung 2013 nicht an die Wirksamkeit dieser Unterstützung geglaubt. Das Problem ist, dass die französische Führung unter Solidarität weniger Deklarationen politischen Beistands versteht, sondern eine konkrete Beteiligung am militärischen Handeln im Sinne Frankreichs. Während die Bundeswehr in Mali an einem eher traditionellen friedenserhaltenden Einsatz der Vereinten Nationen teilnimmt, der das Land stabilisieren soll, zielt der französische Militäreinsatz jedoch außerhalb dieses multilateralen Rahmens auf die Terror- bzw. Aufstandsbekämpfung. Diese fehlende Übereinstimmung verhindert, dass das deutsche Mali-Engagement in Paris in politisches Kapital transformiert werden kann, das wiederum deutschen Interessen in anderen Bereichen zugutekäme. Letztlich läuft die deutsche Politik sogar Gefahr, das Gegenteil von dem zu erreichen, was sie angestrebt hat, sofern sich nämlich in Paris der Eindruck dauerhaft verfestigt, man könne sich auf Berlin nicht verlassen.

2. Münchner Konsens: Anders ist die Wirkung des zweiten Treibers einzuschätzen. Die MINUSMA-Beteiligung unterstreicht glaubhaft die gewachsene Bereitschaft Deutschlands, in der internationalen Politik auch durch ein substantielles Engagement im militärischen Krisenmanagement Verantwortung zu übernehmen. Zwar ließe sich grundsätzlich darüber streiten, ob dieses Politikfeld in besonderer Weise als Ausweis dieser Haltung geeignet ist oder ob nicht andere Formen des sicherheitspolitischen Handelns gleichermaßen oder sogar besser dafür taugen würden. Aber entscheidend ist, dass andere Regierungen die Entsendung von Truppen, die damit verbundenen Kosten und die sich daraus ergebenden Risiken als Ausdruck deutscher Verantwortung und deutschen Gestaltungswillens interpretieren.

Erleichtert wird diese Ergänzung der deutschen UN-Politik dadurch, dass sie sich entlang der tradierten Grundlinien deutscher Sicherheitspolitik bewegt – allgemein, da sie bei der Frage des militärischen Engagements der Bundeswehr multilateralen Prinzipien verpflichtet bleibt, und speziell, da dem MINUSMA-Einsatz die drei konstitutiven Prinzipien für einen Auslandseinsatz der Bundeswehr zugrunde liegen: Er ist international legitimiert durch eine Resolution des UN-Sicherheitsrates; er wird in einem multilateralen Handlungsrahmen durchgeführt; schließlich hat ihn der Bundestag wiederholt durch einen entsprechenden Beschluss mandatiert.

Doch selbst wenn man die These teilt, die Entsendung deutscher militärischer Kontingente sei für das internationale Konfliktmanagement Ausweis größerer internationaler Verantwortung, bleiben zwei grundsätzliche Vorbehalte: Erstens begründet das Argument nicht die Teilnahme an einer spezifischen Mission. So wäre eine Art „Kompensationsgeschäft“ vorstellbar, in dessen Verlauf die deutsche MINUSMA-Beteiligung abgewickelt und durch diejenige an einer anderen *Peacekeeping*-Mission der UN ersetzt wird. Zweitens führt der Wille, zu zeigen, dass man mehr Verantwortung zu tragen bereit ist, nicht zwingend in das Feld friedenserhaltender Missionen der Vereinten Nationen. Ebenso gut könnte Deutschland ihn unter Beweis stellen, indem es größere militärische Lasten im Rahmen der EU oder der NATO übernimmt.

3. Deutschlands Rolle in den Vereinten Nationen: Der deutsche Beitrag zu MINUSMA stärkt die Vereinten Nationen im Bereich der Friedensmissionen militärisch, aber noch viel deutlicher politisch. Damit dient er dem Ziel, das anhaltende Engagement Deutschlands auf diesem Gebiet herauszustellen. Dies geschieht nicht selbstlos, sondern verbindet sich mit der Erwartung, dass Berlin auf die Entscheidungen der Vereinten Nationen mehr Einfluss nehmen kann als ohne ein derartiges Engagement. Ergänzt wird Letzteres durch die erheblichen deutschen Beiträge zur Finanzierung der Friedensmissionen. Aus dieser Sicht ist die deutsche MINUSMA-Beteiligung vor allem als Baustein der deutschen UN-Politik zu begreifen und weniger als Ausdruck einer sicherheitspolitischen Priorisierung, sofern sich diese beiden Politikbereiche überhaupt klar trennen lassen.

4. Europäische Handlungsfähigkeit: Für die Frage, ob mit MINUSMA dieses Ziel erreicht werden kann, ist es wichtig zu unterscheiden, ob man mit der deutschen MINUSMA-Beteiligung die Handlungsfähigkeit der Europäischen Union stärken wollte bzw. will oder diejenige „Europas“. Konkret auf Mali bezogen lässt sich das Argument, MINUSMA stelle einen besonderen Fußabdruck der EU dar, schwer entwickeln, sowohl in institutioneller als auch in quantitativer Hinsicht: Erstens führt die EU in Mali mit der EUTM ihre eigene, anders gelagerte Operation durch, zweitens ist die EU als Institution an MINUSMA nicht beteiligt, drittens gehört aus dem Kreis der EU-Mitglieder lediglich Deutschland zu den zehn größten und damit substantiellen Truppenstellern der Mission, gefolgt von Schweden mit 184 Soldaten, Belgien (53) und Litauen (45). Die restlichen beteiligten EU-Staaten beschränken sich weitgehend auf *Token Contributions* (Stand: 30. November 2021) (United Nations Peacekeeping 2021). Daher kann auch das Argument nicht wirklich verfangen, der Einsatz böte die Möglichkeit, Erfahrungen mit militärischer Interoperabilität zu erwerben und auszubauen. Dies würde in erster Linie für Partner der EU gelten, die sich im Kontext der europäischen „Rückkehr“ zum *Peacekeeping* ebenfalls in Mali engagieren. Lediglich im Fall Großbritanniens mit einem MINUSMA-Kontingent von 255 Soldaten kann dieses Argument überzeugen, dann allerdings mit Bezug auf einen europäischen Partner bzw. ein früheres EU-Mitglied.

Seit Jahren liegen Vorschläge für eine weitergehende autonome operative Rolle der EU im Rahmen des UN-*Peacekeeping* vor, die bereits Eingang gefunden haben in den Plan of Action to Enhance EU CSDP Support to UN Peacekeeping von 2012 sowie die UN-EU Strategic Partnership on Peacekeeping and Crisis Management von 2015. Das Hauptproblem ist jedoch, dass es der EU aufgrund der intergouvernementalen Entscheidungsprozesse nach wie vor schwerfällt, ihre Rolle im internationalen Krisenmanagement auf Dauer zu verstetigen und damit für die Vereinten Nationen Planungssicherheit zu gewährleisten (Rat der Europäischen Union 2018).

5. Regionale Sicherheit: Das vordergründig wichtigste Erfolgskriterium für das deutsche MINUSMA-Engagement wird nicht erfüllt, nämlich der Auftrag der Mission gemäß dem Mandat des UN-Sicherheitsrates – im Gegenteil: Das Friedensabkommen von Algier ist nicht umgesetzt und seit 2015, dem Jahr der Unterzeichnung, hat sich die Sicherheitslage im Land kontinuierlich verschlechtert. Nicht allein dschihadistische Vereinigungen haben diese instabile Lage verursacht, sondern auch lokal geprägte, zum Teil ethnische Konflikte, bewaffnete Milizen und organisierte Kriminalität (Schiller 2020).

Überdies kann keine Rede sein von einer Wiederherstellung der staatlichen Autorität auf dem gesamten Territorium. In Teilen des Landes hat die Regierung die Kontrolle verloren, die Korruption grassiert, es gibt kaum wirtschaftliche Entwicklung. Der Putsch gegen Präsident Ibrahim Boubacar Keïta im August 2020 sowie der neuerliche Umsturz von Übergangspräsident Bah Ndaw und Regierungschef Moctar Ouane im Mai 2021 haben darüber hinaus die Vorstellung ins Wanken gebracht, dass die internationale Gemeinschaft überhaupt Einfluss hätte auf die politischen Entwicklungen in Mali. Entsprechend steht die Frage im Raum, unter welchen Bedingungen MINUSMA und EUTM in Mali weitergeführt werden können (Klatt 2020).

In diesem Sinne kann die bisherige Bilanz der deutschen MINUSMA-Beteiligung nicht als kraftvolles Argument dafür dienen, das deutsche Engagement im UN-*Peacekeeping* fortzusetzen oder gar auszubauen. Schon ein flüchtiger Blick auf andere friedenserhaltende Operationen der UN macht deutlich: Viele dieser Einsätze sind mit exakt den gleichen Herausforderungen konfrontiert. Dabei kann auch der Befund nicht trösten, dass dies kein Spezifikum von UN-Missionen ist, sondern EU- oder NATO-geführte Einsätze ebenso vor dem Problem stehen, dass die angestrebten Erfolge ausbleiben, die lokalen Partner nicht kooperieren und die Missionen scheinbar kein Ende nehmen.

6. Terrorismus und Migration: Schon ein kurzer Blick auf die einschlägigen Indizes lässt Zweifel daran aufkommen, wie groß die Bedrohung tatsächlich ist, die von terroristischen Gruppen aus Mali für Deutschland und Europa ausgeht. So führt der *Global Terrorism Index 2020* Mali nicht unter denjenigen zehn Ländern, die weltweit am stärksten von terroristischen Anschlägen getroffen sind (Institute for Economics & Peace 2020). Zwar sind Mali bzw. der Sahel mittlerweile Teil der „terroristischen Internationale“: Der Islamische Staat in der Großsahara (ISGS), ein neuerer Ableger des „Islamischen Staates“ (IS), verübte 2016 seine ersten Anschläge in Burkina Faso und Niger. Der ISGS hat die Verantwortung für Dutzende Angriffe in Burkina Faso, Mali und Niger übernommen. Indes hat er damit zu kämpfen, eine dominante Präsenz in Burkina Faso, Mali und Niger aufrechtzuerhalten, weil er unter dem Druck von Anti-Terror-Operationen und der Präsenz von Al-Qaida-Verbündeten in der Region steht. Zudem ist vielerorts gar nicht klar, ob hinter den Angriffen tatsächlich Islamisten stecken oder ob es lokale Banditen oder organisierte Verbrecherbanden sind, die ganze Landstriche unter ihre Kontrolle bringen wollen, etwa um Drogen zu schmuggeln. Damit wären nicht so sehr Terroristen das Problem, sondern die kaum funktionierende Staatlichkeit in Mali.

Obendrein erscheint fraglich, ob diese lokalen Gruppen überhaupt die Bereitschaft, viel mehr aber noch die Fähigkeit besitzen, Anschläge in Europa zu verüben. Denn zum einen ist die Migration nach Europa aus einer Vielzahl von Gründen erheblich schwieriger geworden, als es 2015 noch der Fall gewesen sein mag. Seit dem Frühjahr 2020 haben die Beschränkungen durch Covid-19 Reisen nach Europa weitgehend zum Erliegen gebracht.

Desgleichen verblasst das Migrationsargument bei genauerem Hinsehen: Völlig unstrittig ist, dass in Mali lebende Menschen vor der unsicheren Sicherheitslage Schutz suchen. Die daraus resultierende Migration findet jedoch in weit überwiegendem Umfang innerhalb Malis statt bzw. in die umliegenden Länder der Region. Dies deckt sich mit den Zahlen aus den vergangenen Jahren, denen zufolge Personen aus afrikanischen Staaten, die in Deutschland einen Asylantrag gestellt haben, vor allem aus Ländern stammen, die nicht bzw. kaum zur Sahelzone gehören, zum Beispiel Somalia, Eritrea und Nigeria.^7^ Ähnliches gilt für die EU: Vor der Covid-19-Pandemie lag die jährliche Migration aus Mali in die EU bei knapp 20.000 Personen. Selbstverständlich lässt sich keine Auskunft über zukünftige „Migrationswellen“ aus Afrika geben, aber gegenwärtig spielt Mali für Migrationsströme nach Deutschland und in die EU nur eine nachrangige Rolle.

## Ausblick

In der Gesamtschau der in Kap. 4 erfolgten Bewertung ergibt sich ein uneinheitliches Bild. Zwei wesentliche Ziele der Beteiligung an MINUSMA konnten nicht oder nur unzureichend erreicht werden, nämlich den Einsatz für gelebte Bündnissolidarität mit Frankreich zu nutzen und regionale Sicherheit in Mali bzw. der Sahelzone zu gewährleisten. Auch als Ausweis europäischer Handlungsfähigkeit taugt er nur bedingt. Unter sicherheitspolitischen Gesichtspunkten ist die Mission also ein klarer Fehlschlag. Hingegen geht das starke und wirksame Signal von ihm aus, dass Deutschland das UN-System unterstützt und Verantwortung übernimmt, um den Multilateralismus zu erhalten. In dieser Hinsicht lässt sich der Einsatz durchaus als Erfolg lesen. Als scheinbar paradoxes Fazit ließe sich formulieren, der deutsche MINUSMA-Einsatz „ist erfolgreich, ohne erfolgreich zu sein“. Denn mit Blick auf das skizzierte Bündel von Motiven, das Deutschland veranlasst hat, sich an MINUSMA zu beteiligen, muss konstatiert werden, dass einige, aber eben nicht alle Ansprüche verwirklicht werden konnten (Patz 2019, S. 357–358).

Die Erfahrung, dass mit Truppenentsendungen zu UN-Missionen nicht alle angestrebten Ziele gleichermaßen zu erreichen sind, liegt in der Multidimensionalität solch politisch komplexer Stationierungsentscheidungen begründet:

Entscheidungen darüber, ob und was beigetragen werden soll, sind daher am besten als Produkte eines Wettbewerbs zwischen motivierenden und hemmenden Faktoren zu verstehen, dessen Ergebnis von den politisch Verantwortlichen bestimmt wird, die durch den jeweiligen Kontext mit einer begrenzten Anzahl von Optionen, mit Präferenzen und erwarteten Vorteilen konfrontiert werden (Bellamy und Williams 2013, S. 418, eigene Übersetzung).

In diesem Spannungsfeld sind grundsätzlich vier Entwicklungspfade für die deutsche Politik vorstellbar. Zwar lassen sich ihre jeweiligen Vor- und Nachteile wissenschaftlich erfassen, aber letztlich muss die Politik über sie entscheiden:

1. Die „isolationistische“ Option: Nach den enttäuschenden Ergebnissen von MINUSMA in Mali und der Sahel-Region würde sich Deutschland vollständig aus dem UN-*Peacekeeping* zurückziehen, entweder sofort oder nach Ende der Mission. Da die meisten der geschilderten Probleme beim Krisenmanagement ebenso auf derartige Einsätze der NATO bzw. der EU zutreffen können, müsste die Bundeswehr diesen Schwenk konsequenterweise auch in diesem Rahmen vollziehen und sich fortan ausschließlich auf Bündnis- und Landesverteidigung konzentrieren. Dies würde umso leichter fallen, als in der nordatlantischen Allianz die Ära der großformatigen Stabilisierungseinsätze ohnehin zu Ende geht. Der Reputation Deutschlands als Stütze des Multilateralismus und als verantwortungsvolle Mittelmacht in der internationalen Politik würde jedoch schwerer Schaden zugefügt. Letztlich würde Deutschland das System schädigen, das die Vorbedingung von Sicherheit und Prosperität bildet und von dem es selbst am meisten profitiert.

2. Die „symbolische“ Option: Mit dieser Option würde die Bundesrepublik zur Frühphase ihres Engagements im UN-*Peacekeeping* zurückkehren und sich wieder auf symbolische Beiträge beschränken, das heißt auf Truppenstellungen, die ihr Engagement in einem Krisengebiet politisch unterstreichen sollen, aber nicht auf militärische Wirksamkeit abzielen. Mit dieser Politik würde Deutschland weiterhin die UN als wichtigste Organisation für Frieden und Sicherheit politisch zu stärken suchen, aber keinen wirklichen Beitrag zu deren militärischer Effizienz leisten. Die Möglichkeiten, mit europäischen Verbündeten zu kooperieren, wären begrenzt.

3. Die „Status quo“ Option: In diesem Fall würde Deutschland den eingeschlagenen Weg eines größeren Engagements im UN-*Peacekeeping* fortsetzen, und zwar mit nationalen Beiträgen auf gleichbleibendem Niveau und in der Erwartung, damit verschiedene außenpolitische Ziele auf einmal erreichen zu können, etwa den Multilateralismus zu stärken sowie Krisen und Konflikte einzuhegen. Durch institutionalisierte Kooperation mit europäischen Partnern würde zugleich die Handlungsfähigkeit Europas in der internationalen Politik ausgebaut.

4. Die „intensivierte“ Option: Gerade aufgrund der mäßigen Erfolge von MINUSMA würde Deutschland mit dieser Option sein Engagement im UN-*Peacekeeping* erhöhen, auch militärisch. Ziel wäre hier, die Vereinten Nationen auf Dauer mit deutschen und europäischen Beiträgen zu einem effektiven Anbieter von Sicherheit zu entwickeln. In der Folge würden friedenserhaltende Maßnahmen der UN zu einem Schwerpunkt deutscher Sicherheitspolitik, an dem sich alle militärischen Planungen und Beschaffungsvorhaben auszurichten hätten.

Unabhängig von der jeweils bevorzugten Option ist mehr strategische Klarheit in dieser Frage notwendig – gleichermaßen mit Blick auf die deutsche Innenpolitik wie auf internationale Partner. Die Ziele deutscher Beiträge zu UN-geführten Friedenseinsätzen sind strategisch nach wie vor unterreflektiert. Dementsprechend bleiben viele Bekundungen vage und leisten einem Ad-hoc-Vorgehen Vorschub. Ein Strategiepapier der Bundesregierung zu deutschen UN-Kontingenten würde die Brücke zwischen deutscher UN-Politik und deutscher Sicherheitspolitik schlagen, den operativen Planungen Kontinuität und Partnern Planungssicherheit geben. Dabei müsste den gewandelten Anforderungen an das *Peacekeeping* einerseits und der veränderten Struktur des internationalen Systems andererseits Rechnung getragen werden, zum Beispiel bei der Auswahl von Partnern. Die Erarbeitung des Strategiepapiers sollte auf die laufenden Prozesse zur Erstellung des Strategischen Kompasses der EU bzw. des neuen strategischen Konzepts der NATO abgestimmt werden. Deutsche UN-Kontingente werden nämlich noch stärker als bisher multinationales Handeln widerspiegeln. Eine entsprechende Priorisierung in den Teilen dieser beiden Schlüsseldokumente, die sich mit Krisenmanagement beschäftigen, würde die Grundlage dafür legen. In diesem Zusammenhang wird der Frage von Partnerschaften und derjenigen der Arbeitsteilung zwischen den globalen Sicherheitsinstitutionen besondere Bedeutung zukommen.

## Footnotes

1 Im vorliegenden Artikel wird, abweichend vom ZfAS-Standard, bei personenbezogenen Substantiven die männliche grammatikalische Form verwendet. Der Autor schließt damit Personen jeden Geschlechts gleichermaßen ein.

2 Diese häufig anzutreffende Form der Beteiligung an UN-Friedenssicherungseinsätzen kommt in drei Varianten vor: 1) Stabsoffiziere, die in operativen Hauptquartieren arbeiten; 2) Militärbeobachter; 3) kleinere Truppenbeiträge, die in größere militärische Kontingente anderer Nationen integriert werden (vgl. Coleman 2013).

3 Vgl. zu dieser Grundhaltung des „opportunistischen Multilateralismus“ Lacher (2021, S. 26–27).

4 Vgl. dazu die Äußerungen eines Referatsleiters in der Abteilung Politik, Bundesministerium der Verteidigung (zit. n. Hilpert 2014, S. 182, eigene Übersetzung): „Wir sind nicht in erster Linie in Mali wegen Mali. Wir sind in erster Linie in Mali, weil wir ein Interesse an der Zusammenarbeit mit Frankreich haben, weil wir ein Interesse daran haben, dass die EU ein funktionierender sicherheitspolitischer Akteur ist, und weil wir ein Interesse daran haben, dass die UNO ein Akteur im internationalen Konfliktmanagement ist. Und erst in zweiter Linie, nach diesen Faktoren, war es unser Motiv, uns an einem Militäreinsatz zu beteiligen, war unser Interesse, dass Mali stabil ist und Frieden findet.“.

5 Vgl. zur Rezeption dieser Reden ausführlich die Beiträge in Hellmann et al. (2015).

6 Aus der Fülle der Literatur zu Multilateralismus sei nur verwiesen auf Maull (2020), Benner (2019) sowie Heemann und Rosenow (2019).

7 Vgl. dazu die Zahlen des Bundesamtes für Migration und Flüchtlinge aus den Jahren 2014 bis 2021 (Bundeszentrale für politische Bildung 2021).
